# Tranexamic Acid Use Intra-Operatively Decreases the Need for Blood Transfusions and Post-Operative Edema in Temporomandibular Joint Surgeries

**DOI:** 10.7759/cureus.31569

**Published:** 2022-11-16

**Authors:** Bayan Entezari, Larry M Wolford, Daniel C Gunn, Sergio Murillo, Saravanan Ramamoorthy

**Affiliations:** 1 Medicine, Texas A&M College of Medicine, Dallas, USA; 2 Department of Oral and Maxillofacial Surgery, Baylor University Medical Center, Dallas, USA; 3 Department of Anesthesiology, Baylor University Medical Center, Dallas, USA; 4 Anesthesiology and Perioperative Medicine, U.S. Anesthesia Partners, Dallas, USA

**Keywords:** tranexemic acid, edema, oral and maxillofacial surgery, intraoperative blood loss, edema score

## Abstract

Temporomandibular joint (TMJ) surgeries cover a vast assortment of surgical procedures such as tooth extractions, tissue biopsies, and extensive maxillofacial surgeries. Major complications that occur during and after TMJ surgeries include uncontrolled bleeding, considerable blood loss, serious infections, and edema. Tranexamic acid (TXA) is an antifibrinolytic agent that reduces blood loss by inhibiting the enzymatic breakdown of fibrin. Currently, TXA is widely used in various orthopedic surgeries to reduce bleeding and decrease the need for blood transfusions. In this study, we observed five patients undergoing major TMJ replacement surgeries and administered TXA during the procedure. The principal aim of this study was to examine the association between TXA administration during TMJ replacement surgery and blood loss and tissue edema.

## Introduction

Temporomandibular joint (TMJ) replacement surgeries are often associated with significant intraoperative blood loss (up to 1 liter of blood volume) and often require blood transfusion [1,2]. Intraoperative bleeding during total TMJ replacements can occur due to the proximity of major vessels such as the maxillary artery, temporal artery, and facial artery [3]. Large amounts of blood loss can cause hemodynamic instability, leading to a requirement for blood transfusions [4]. Blood transfusions often carry risks such as immunologic reactions, blood-borne infections, lung injuries, post-operative edema, and in severe cases, even death [5]. Post-operative edema is evaluated using an established edema score in orthognathic surgery literature which ranges from grades I-IV. The edema score is determined through visualization with a score of I being slight edema (hardly visible), II being mild edema, III being moderate swelling within the application area, and IV being severe swelling beyond the application area. Close monitoring of edema is essential in the post-operative phase of TMJ replacement surgeries because of its proximity to the airway [6]. Additionally, the presence of severe and prolonged post-operative edema leads to delayed wound healing and distortion of TMJ anatomy.

Tranexamic acid (TXA) has been shown to limit blood loss and decrease the need for transfusions in various orthopedic and orthognathic surgeries. TXA is an antifibrinolytic agent which competitively inhibits the activation of plasminogen to plasmin; it also stops the degradation of fibrin clots and preserves platelets for clot formation [4]. A number of studies have suggested the anti-inflammatory characteristics of TXA by inhibiting plasmin-mediated activation of complement, monocytes, and neutrophils [7]. At therapeutic levels, TXA is safe without directly affecting platelets or coagulation factors [8]. TXA has a half-life of about two hours and is excreted unchanged by the kidneys [9]. This case report aimed to examine the role of TXA in minimizing blood loss, decreasing the need for blood transfusion, and decreasing post-operative edema during TMJ surgeries.

## Case presentation

The cases included in this study consist of five patients who underwent TMJ replacement surgeries from January 2021 to June 2021 at Baylor University Medical Center (BUMC). Data was recorded for each patient before, during, and after the TMJ replacements. In our population, the propensity of TMJ disorder was five times more in female patients than in male patients, therefore all patients included in this case report were female. Pre-operative hemoglobin was recorded in all patients prior to TXA infusions. All patients received 10 mg of dexamethasone. Additionally, all patients were administered a 10 mg/kg bolus dose of TXA prior to incision, followed by a continuous infusion of 1 mg/kg/hr intraoperatively. Table 1 summarizes the blood loss, pre-and post-operative hemoglobin levels, blood products given, and post-operative edema scores assessed on postoperative day 1. None of the patients required intra-operative or post-operative blood transfusions.

**Table 1:** Patient Recorded Information pRBC: packed red blood cells; TXA: tranexamic acid.

Age	TXA	Estimated blood loss	Pre-Op diagnosis	pre-op hgb	post-op hgb	pRBC given intraop	pRBC given post-op	Edema Score (I-IV)
39F	Tranexamic acid 1,000 mg/10 mL intravenous solution → 800 mg Tranexamic acid 1,000 mg in 0.9% sodium chloride (NaCl) 90 mL infusion→ 612 mg	350 ml	1. Rheumatoid arthritis of temporomandibular joint 2. Mandibular hypoplasia	12.9	11.1	none	none	I
40F	tranexamic acid 1,000 mg in 0.9% sodium chloride (NaCl) 50 mL → 650 mg tranexamic acid 1,000 mg in 0.9% sodium chloride (NaCl) 100 mL infusion → 393.7 mg	400 ml	1. Bilateral TMJ arthritis 2. Maxillary hypoplasia 3. Mandibular hypoplasia 4. Bilateral hypertrophied turbinates	13.2	10.4	none	none	II
63F	tranexamic acid 1,000 mg in 0.9% sodium chloride (NaCl) 50 mL → 600 mg tranexamic acid 1,000 mg in 0.9% sodium chloride (NaCl) 100 mL infusion → 354 mg	100 ml	1. Bilateral TMJ arthritis 2. Mandibular hypoplasia 3. Maxillary hypoplasia	12.7	10.4	none	none	II
63F	Tranexamic acid 1,000 mg/10 mL intravenous solution → 650 mg tranexamic acid 1,000 mg in 0.9% sodium chloride (NaCl) 100mLinfusion→ 574.88 mg	300 ml	1. Bilateral TMJ arthritis 2. Maxillary hypoplasia 3. Mandibular hypoplasia 4. Right calcified stylohyoid ligament	13.2	10.1	none	none	I
39F	tranexamic acid 1,000 mg in 0.9% sodium chloride (NaCl) 50 mL → 1,000 mg tranexamic acid 1,000 mg in 0.9% sodium chloride (NaCl) 100 mL infusion → 490.63 mg	350 ml	1. Bilateral TMJ arthritis. 2. Maxillary hypoplasia. 3. Mandibular hypoplasia. 4. Bilateral hypertrophied turbinates 5. Calcified stylohyoid ligaments	12.7	9.6	none	none	II

The first patient as shown in Table 1 was a 39-year-old female with a past medical/surgical history of three prior jaw surgeries and three prior nasal septum surgeries. Her pre-operative diagnosis consisted of rheumatoid arthritis of the TMJ and mandibular hypoplasia. The procedures she underwent included reconstruction with a total joint prosthesis, bilateral TMJ fat grafts harvested from the abdomen, bilateral coronoidectomies, and maxillary osteotomy with bone plate stabilization and bone grafting. Her pre-operative hemoglobin was 12.9. Following surgery, her post-operative hemoglobin ended up being 11.1 and she had an edema score of I. This patient received a total of 1,412 mg of TXA and had a blood loss of 350 ml intra-operatively. The rest of her post-operative course was uneventful.

The second patient listed in Table 1 was a 40-year-old female with a past medical/surgical history of brain surgery, foot surgery, hysterectomy, tonsillectomy, and depression. Her pre-operative diagnosis consisted of bilateral TMJ arthritis, maxillary hypoplasia, mandibular hypoplasia, and bilateral hypertrophied turbinates. This patient underwent TMJ reconstruction with a total joint prosthesis, bilateral TMJ fat grafts harvested from the abdomen, bilateral coronoidectomies, maxillary osteotomy with bone plate stabilization and bone grafting, and partial inferior turbinectomy. For this patient, the pre-operative hemoglobin was 13.2. Post-operatively, her hemoglobin dropped to 10.4 and she had an edema score of II. This patient received a total of 1,043.7 mg of TXA and had a blood loss of 400 ml intra-operatively. Post-surgery, this patient progressed slowly and had difficulty with pain management. Additionally, she developed hypokalemia but recovered and was discharged safely within a couple of days.

The third patient listed in Table 1 was a 63-year-old female with a past medical/surgical history of collagenous colitis, deviated nasal septum, endometriosis, fibromyalgia, gastroesophageal reflux disease (GERD), hypothyroidism, migraines, osteoarthritis, obstructive sleep apnea, prior C-section, and hysterectomy. Her pre-operative diagnosis was bilateral TMJ arthritis, mandibular hypoplasia, and maxillary hypoplasia. She underwent TMJ replacement, maxillary osteotomies, fat grafts, coronoidectomies, and oral splints. Her pre-operative hemoglobin was 12.7 which dropped to 10.4 post-operatively. Her post-operative edema score was II. This patient received a total of 954 mg of TXA and had a blood loss of 100 ml intra-operatively. The rest of her post-operative course included difficulties with pain management, nausea, and vomiting. The patient then developed hypokalemia on postoperative day 3, requiring management with potassium protocol which resolved on day 5. She also developed a reaction to Norco that caused a severe migraine headache on day 7, delaying discharge. The patient significantly improved before being discharged.

The fourth patient listed in Table 1 is another 63-year-old female with a past surgical history of tonsillectomy and hysterectomy. Her pre-operative diagnosis consisted of bilateral TMJ arthritis, maxillary hypoplasia, mandibular hypoplasia, and right calcified stylohyoid ligament. She underwent TMJ reconstruction with a total joint prosthesis, bilateral TMJ fat grafts harvested from the abdomen, multiple maxillary osteotomies with bone plate stabilization and bone grafting, anterior mandibular horizontal osteotomy, and an application of maxillary and mandibular arch bars and bone screw fixation with 10 bone screws. Her pre-operative hemoglobin was 13.2. Post-operatively, her hemoglobin was 10.1 and she had an edema score of I. This patient received a total of 1,224.88 mg of TXA and had a blood loss of 300 ml intra-operatively. The rest of her post-operative course was uneventful.

The final patient listed in Table 1 was another 39-year-old female with a past medical history of anxiety, chronic pain, depression, mild heart murmur, hypothyroidism (resolved), and sleep apnea. This patient’s pre-operative diagnosis consisted of bilateral TMJ arthritis, maxillary hypoplasia, mandibular hypoplasia, bilateral hypertrophied turbinates, and calcified stylohyoid ligaments. She underwent TMJ reconstruction with a total joint prosthesis, bilateral TMJ fat grafts harvested from the abdomen, bilateral resection and removal of calcified stylohyoid ligaments, maxillary osteotomies with bone plate stabilization, anterior mandibular horizontal ostectomy, and bilateral partial inferior turbinectomy. Her pre-operative hemoglobin was 12.7. Post-operatively, her hemoglobin was 9.6 and she had an edema score of II. This patient received a total of 1,490.63 mg of TXA and had a blood loss of 350 ml intra-operatively. This patient’s post-operative course consisted of difficulties with finding a compatible pain medication. She also had difficulties with urinary retention which required repeated catheterizations. Additionally, she had a significant reaction to bethanechol. Eventually, Urology had to be consulted for urinary issues. The patient improved and was discharged.

## Discussion

This case report aimed to examine the association between TXA administration during TMJ replacement surgery, blood loss, and tissue edema. TXA has been routinely used during most orthopedic and trauma surgeries to limit blood loss [10]. While the benefit of TXA is well established in orthopedic procedures such as hip and knee replacement, its benefit in TMJ replacement surgeries remains largely unknown. The CRASH-2 study involving 20,211 adult trauma patients, who were at risk for significant hemorrhage, showed a significant reduction in mortality without any significant increase in thromboembolic events [11]. As this is a case report, a true comparison could not be obtained. However, historical averages of estimated blood loss and blood transfusions at BUMC of similar surgeries performed by the same surgical team were obtained. The average blood loss where TXA was not used was around 800 ml during TMJ surgeries performed by the same surgical team. The length of surgeries was not able to be obtained. In the cases included in this report, all patients were administered a 10 mg/kg bolus dose of TXA prior to incision, followed by a continuous infusion of 1 mg/kg/hr intraoperatively. The dosing of TXA, used in these cases, was based on findings from studies in cardiac surgery that noted a 10 mg/kg loading dose followed by a TXA infusion of 1 mg/kg/hr produces plasma concentrations sufficient to inhibit fibrinolysis, and that a larger dose does not provide additional hemostatic benefit [11].

The range of estimated blood loss in these cases was 100-400 mL. Blood loss was estimated through visual estimation. In a meta-analysis study involving 26 studies and 3,297 patients, it was found that visual estimation was the most used technique [12]. Advocates of visual estimation note its intuitive ease and that, in the context of appropriate supporting laboratory and vital sign metrics, further granularity rarely provides additional short-term clinical insight [12]. Blood loss in these cases was estimated in a similar fashion to historical cases at our institution, which is done by counting the number of blood-soaked sponges, visual inspection of the surgical field, and the amount of blood in the suction canisters. Furthermore, no drains were placed in any patients for these procedures.

Additionally, in this case report, none of the patients required any blood transfusions either intra-operatively or post-operatively. The post-operative edema score was II or lower in all patients who received TXA. Historically, in similar cases performed by the same surgical team at BUMC where TXA was not administered, an edema score of III and higher was usually seen. The anti-inflammatory properties of TXA could also be contributing to lower post-operative edema scores in these surgeries.

Despite the potential benefits of TXA identified in this case report, limitations of the study should be recognized. The number of patients included in this case report is small due to the nature of the quality initiative. The effect of TXA on post-operative edema would be better delineated with a larger number of participants and by using tissue edema infra-red camera as opposed to a visual assessment of edema. Even though TXA has been used in patients routinely, this case report population did not show any apparent adverse effects such as thromboembolic events, visual disturbances, and hypersensitive reactions [13]. The side effect profile might be different in a larger study population.

## Conclusions

In summary, five female patients of varying ages received TXA intra-operatively during TMJ replacement surgery. Even though all patients had a decrease in postoperative hemoglobin levels, none of them required blood transfusion. An edema score of II and below were seen in this study population. TXA’s ability to decrease blood loss reduces the need for blood transfusions. A decrease in fluid shifts along with the anti-inflammatory properties of TXA could decrease post-operative edema and improve wound healing. 

## References

[1] Carls FR, Engelke W, Locher MC (1996). Complications following arthroscopy of the temporomandibular joint: analysis covering a 10-year period (451 arthroscopies). J Craniomaxillofac Surg.

[2] Maalouly J, El Assaad D, Ayoubi R (2020). Efficacy and safety of systemic tranexamic acid administration in total knee arthroplasty: a case series. Int J Surg Case Rep.

[3] Hoffman D, Puig L (2015). Complications of TMJ surgery. Oral Maxillofac Surg Clin North Am.

[4] Rohrich RJ, Cho MJ (2018). The role of tranexamic acid in plastic surgery: review and technical considerations. Plast Reconstr Surg.

[5] (2006). Practice guidelines for perioperative blood transfusion and adjuvant therapies: an updated report by the American Society of Anesthesiologists Task Force on Perioperative Blood Transfusion and Adjuvant Therapies. Anesthesiology.

[6] Kumar JN, Ravi P (2021). Postoperative Care of the Maxillofacial Surgery Patient. Oral and Maxillofacial Surgery for the Clinician.

[7] Robertshaw HJ (2008). An anti-inflammatory role for tranexamic acid in cardiac surgery?. Crit Care.

[8] Chang R, Holcomb JB, Leibner E, Pommerening M, Kozar RA (2019). Hemostasis, surgical bleeding, and transfusion. Schwartz's Principles of Surgery.

[9] Mielke RT, Obermeyer S (2020). The use of tranexamic acid to prevent postpartum hemorrhage. J Midwifery Womens Health.

[10] Roberts I (2015). Tranexamic acid in trauma: how should we use it?. J Thromb Haemost.

[11] Williams-Johnson JA, McDonald AH, Strachan GG, Williams EW (2010). Effects of tranexamic acid on death, vascular occlusive events, and blood transfusion in trauma patients with significant haemorrhage (CRASH-2): a randomised, placebo-controlled trial. West Indian Med J.

[12] Tran A, Heuser J, Ramsay T, McIsaac DI, Martel G (2021). Techniques for blood loss estimation in major non-cardiac surgery: a systematic review and meta-analysis. Can J Anaesth.

[13] Nugent M, May JH, Parker JD (2019). Does tranexamic acid reduce knee swelling and improve early function following arthroscopic meniscectomy? A double-blind randomized controlled trial. Orthop J Sports Med.

